# The Unknown Abnormal Condition Monitoring Method for Pumped-Storage Hydroelectricity

**DOI:** 10.3390/s23146336

**Published:** 2023-07-12

**Authors:** Jun Lee, Kiyoung Kim, Hoon Sohn

**Affiliations:** Department of Civil and Environmental Engineering, Korea Advanced Institute of Science and Technology, Yuseong-gu, Daejeon 34141, Republic of Korea

**Keywords:** continuous learning, condition monitoring, prognostics and health management, pumped-storage hydroelectricity

## Abstract

Pumped-storage hydroelectricity (PSH) is a facility that stores energy in the form of the gravitational potential energy of water by pumping water from a lower to a higher elevation reservoir in a hydroelectric power plant. The operation of PSH can be divided into two states: the turbine state, during which electric energy is generated, and the pump state, during which this generated electric energy is stored as potential energy. Additionally, the condition monitoring of PSH is generally challenging because the hydropower turbine, which is one of the primary components of PSH, is immersed in water and continuously rotates. This study presents a method that automatically detects new abnormal conditions in target structures without the intervention of experts. The proposed method automatically updates and optimizes existing abnormal condition classification models to accommodate new abnormal conditions. The performance of the proposed method was evaluated with sensor data obtained from on-site PSH. The test results show that the proposed method detects new abnormal PSH conditions with an 85.89% accuracy using fewer than three datapoints and classifies each condition with a 99.73% accuracy on average.

## 1. Introduction

Pumped-storage hydroelectricity (PSH) is a hydroelectric energy storage method used for load balancing in hydroelectric power plants [1,2]. When the electricity consumption on the electrical grid is low, PSH stores energy as the gravitational potential energy obtained by pumping water from a lower to a higher elevation reservoir. This operational state is known as a pumped state and is commonly used in conventional hydroelectricity. In contrast, when the electricity consumption is extremely high, water from an upper reservoir is carried downhill and drives a hydropower turbine and generator to produce electricity to meet the increased demand, which is called the turbine state. One advantage of a PSH system is that its output power regulation is simpler because there is a shorter transition time between its operation and shutdown. Consequently, PSH can maintain the constant frequency and voltage of generated power with relative ease. 

However, PSH is a complex and nonstationary system, in which many components influence and interact with other components. A hydropower turbine, one of the primary power-generating components of PSH, experiences various time-varying loads during its operation. These loads can induce local, cyclic, and thermal stresses in the rotating system in operation, which frequently cause abnormal conditions in main facilities. As listed in Table 1, typical abnormal conditions include steel corrosion, steel wear, fatigue stress, cracks, bolt loosening, over vibration, and overheating [3,4,5]. These abnormal conditions should be closely examined during operation, as they can induce power generation efficiency decreases in the facilities of a PSH system.

Monitoring the accidental occurrence of these abnormal conditions is usually challenging because the primary components of PSH, such as a hydropower turbine and generator, are submerged in water during operation. To tackle this issue, long-term condition-monitoring systems have been constructed with several types of sensors attached to the exterior surfaces of the primary components. By collecting long-term physical responses and analyzing the potential abnormalities of the components, these monitoring systems allow for targeted maintenance and emergency planning [6]. Due to the definite benefit, abnormal condition diagnoses using these monitoring systems are gaining recognition as being crucial to the operation and maintenance of the primary components of PSH [7,8,9].

A series of studies have been conducted on the abnormal condition monitoring of PSH systems, and one of the most popular and important topics is vibration signal analyses of hydraulic turbines [7,8,10]. These signal analyses allow for a rapid diagnosis of hydraulic turbine failures. Furthermore, a novel nonlinear modeling methodology for hydropower generation systems was proposed considering the vibration characteristics and pipe flow in a hydraulic turbine [11]. The performance of the model was verified by comparing it with actual data acquired from a structure, and abnormal conditions could be detected with a high accuracy and fast calculation times. However, the aforementioned condition-monitoring methods only detect abnormal magnitudes or frequencies of single-sensor measurements instead of considering all the sensor measurements to diagnose the system. Hence, the types of abnormal conditions that can be detected by these methods are limited to predefined ones, and the spatial range of the condition monitoring is also limited to the specific components of the system.

To overcome these limitations, machine learning techniques such as clustering [12], support vector machine classifiers [13,14], and random forest [15] have been widely applied to the fault diagnosis and condition monitoring of PSH systems. Introducing machine learning enables the use of a large number of sensors for a complex signal analysis and monitoring not only the physical responses of the major components, but also the overall structural movement of the system. However, because several sensors are installed in a typical system, machine-learning-based condition-monitoring methods require significant time to train their models and classify the conditions of a target structure. Another shortcoming of the current machine-learning-based methods is that an abnormal condition not included in the training dataset is inevitably classified as an incorrect abnormal class. The model must be retrained with expert involvement to learn newly recognized abnormal classes. 

In this study, a condition-monitoring method is proposed for the end-to-end intelligent diagnosis of PSH. The proposed method employs two algorithms: (1) a new class recognition algorithm that detects a novel type of abnormal condition that is not trained with a dataset, and (2) a continuous learning algorithm that trains and optimizes a previously trained model without human involvement when detecting a new type of abnormal condition. Using these two algorithms, the proposed method provides a high level of accuracy in the recognition of new types of abnormal conditions and the classification of automatically trained models.

The proposed method offers the following advantages: (1) monitoring and classifying the overall state of the structure using various sensor data, (2) detecting unknown abnormal conditions using less than three datapoints without retraining or modifying the trained model, and (3) automatically optimizing the architecture of the classification model for a new abnormal class.

The remainder of this paper is organized as follows. Section 2 presents a detailed description of the proposed condition-monitoring method based on continuous learning. The experimental validation is presented in Section 3, and Section 4 concludes this study. 

## 2. Pumped-Storage Hydroelectricity Condition-Monitoring Method

**Figure 1:**
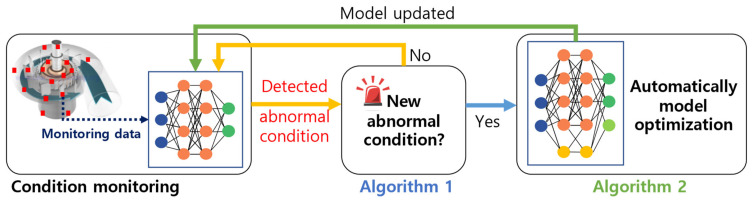
Flowchart of the proposed condition-monitoring method. The proposed method consists of Algorithm 1, class detection network of novelty class based on Euclidean distance in feature map, and Algorithm 2, automatic model reconstruction and optimization for new class using dynamic expansion network.

Section 2 describes the condition-monitoring procedure of the proposed method. Figure 1 shows a flowchart of the proposed condition-monitoring method based on the auto-learning and class detection network (ACDN) model. As shown in Figure 1, the proposed method consists of two algorithms: (1) a class detection network of novelty classes based on the Euclidean distance in feature maps, and (2) automatic model reconstruction and optimization for new classes through dynamic expansion networks, denoted as Algorithm 1 and 2, respectively. Based on the results of processing the monitoring data with the pretrained model, Algorithm 1 determines if the monitoring data correspond to an existing learned or new class. When a new class is identified by Algorithm 1, Algorithm 2 extends and optimizes the pretrained model for an increased number of classes.

### 2.1. Base Model Configuration of ACDN

**Figure 2:**
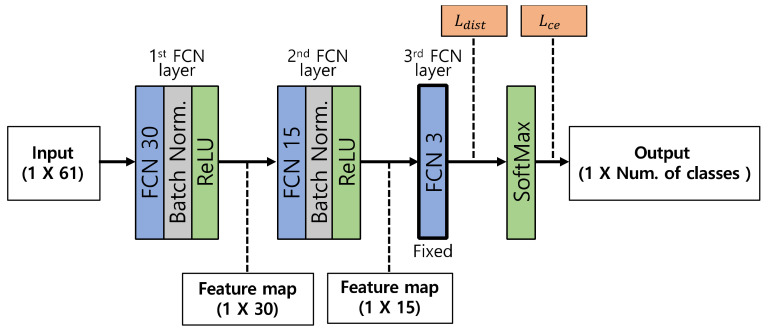
Auto-learning and class detection network model configuration.

To classify the conditions of a target structure, the ACDN builds a one-dimensional Fully Connected Network (FCN), as shown in Figure 2. The input data consist of 61 sensor monitoring data and are structured in the form of a one-dimensional vector. The ACDN has three FCN layers. The first and second FCN layers consist of two distinct FCN layers with batch normalization and a rectified linear unit (ReLU) activation function. The first and second FCN layers initially have 30 and 15 neurons, respectively. The number of neurons can be modified using the auto-learning optimization of Algorithm 2. The last FCN consists of three neurons that calculate the loss of class distance $L_{dist}$, which is computed and used in Algorithm 1 to detect a new abnormal condition by minimizing the intraclass distance and maximizing the interclass distance. The output vector is calculated through the SoftMax layer with logit vectors from the last FCN layer. In ACDN training, $L_{dist}$ and the cross-entropy loss $L_{ce}$ converge to a minimum. 

### 2.2. Algorithm 1: Open Set Recognition for Detecting New Abnormal Conditions

**Figure 3:**
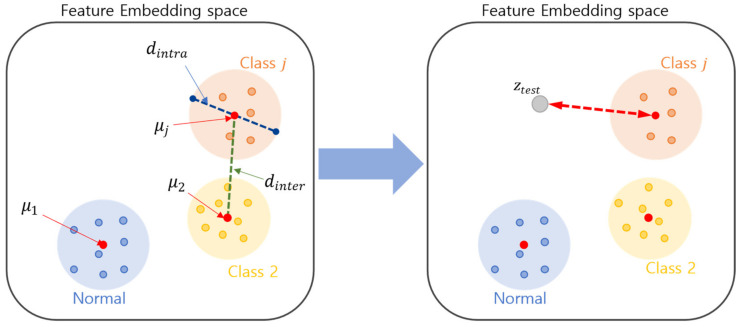
Overview of unknown class detection methods.

In a typical neural network classifier, the activation vector computed from the final fully connected layer is first applied as an input to a SoftMax activation function. Afterward, the network is trained to minimize a loss function such as the cross-entropy on the outputs of the SoftMax layer. In Algorithm 1, the activation vector z of the final fully connected layer is the projection of the input vector x (i.e., $z=g\left(x\right)$) onto a different space, as shown in Figure 3. z is applied to the ii-loss algorithm that maximizes the distance between the different classes (i.e., interclass distance) while minimizing the distance between the data within the class (i.e., intraclass distance). 

Consider that c classes have been classified and the number of input data in the jth class is $n_{j}$. The input data are structured as an input vector $x_{i}$ and the activation vector $v_{i}$ is generated through the final fully connected layer. Then, the intraclass distance $d_{intra}^{j}$ of the jth class is calculated, using (1) as

(1) $$d_{intra}^{j}=\frac{1}{n_{j}}\sum_{i=1}^{n_{j}}||\mu_{j}-{\left.v_{i}\right||}_{2}^{2}$$

where $\mu_{j}$ is the mean of the activation vector of class j:

(2) $$\mu_{j}=\frac{1}{n_{j}}\sum_{i=1}^{n_{j}}v_{i}$$

The average intraclass distance of all c classes are calculated, using (3) as

(3) $$d_{intra}=\frac{1}{c}\sum_{j=1}^{c}d_{intra}^{j}$$

The interclass distance $d_{inter}$ is calculated in terms of the distance between the mean of the two classes among all the K classes as

(4) $$d_{inter}=\underset{\begin{array}{c} 1\leq a\leq c\\ a+1\leq b\leq c \end{array}}{\min}||\mu_{a}-{\left.\mu_{b}|\right|}_{2}^{2}$$

The network is trained with stochastic gradient descent with backpropagation to minimize the loss function $L_{dist}$ defined in (5), because minimizing $L_{dist}$ indicates minimizing $d_{intra}$ and maximizing $d_{inter}$ as

(5) $$L_{dist}=d_{intra}-d_{inter}$$

After the network training is complete, $\mu_{j}$ and $d_{intra}^{j}$ are calculated for each class with all the training instances for that class and are stored as part of the model. 

During testing, an outlier score function $OS\left(x_{test},j\right)$ quantifies the degree of input data $x_{test}$ to be predicted as an outlier for the classes. The outlier score is calculated as the distance between the activation vector $z_{test}=g\left(x_{test}\right)$ and the $\mu_{j}$ of the closest class.

(6) $$OS\left(x_{test},j\right)=\underset{1\leq j\leq K}{\min}||\mu_{j}-{\left.z_{test}|\right|}_{2}^{2}$$

Because the network is trained to project the class members as close as possible to the mean of the class, the further the projection $z_{test}$ of the input data $x_{test}$ is from the mean of its closest class, the greater the possibility that the instance is an outlier for the class.

After identifying an appropriate outlier score for the existing classes, the threshold value of the outlier score is determined to distinguish a new condition. Even under normal conditions, outlier data can be generated intermittently in PSH because of measurement or signal transmission errors. On the feature map in the ACDN, the distance between the outlier data and $\mu_{1}$ was calculated and compared with $d_{intra}^{1}$, where the normal condition of c is set as 1. The outlier data were more than 1.16 times the $d_{intra}^{1}$. Therefore, the threshold value, ε, is set to 1.16 to identify a new abnormal condition. The outlier data were only measured on an irregular basis, distinguishing them from abnormal conditions. Therefore, when the $OS\left(x_{test},j\right)$ of three or more consecutive data points are large, based on (7), it is determined to be a new class.

(7) $$OS\left(x_{test},j\right)>\varepsilon d_{intra}^{j}$$

### 2.3. Algorithm 2: Model Optimization for Adding New Abnormal Condition

If Algorithm 1 identifies a new abnormal condition, Algorithm 2 optimizes the ACDN using a continuous learning method without human involvement. Consider that the number of conditions (i.e., the total number of classes including normal and abnormal conditions) increases from c−1 to c after the execution of Algorithm 1 and the training data that correspond to c classes are $D_{c}={\left\{x_{i},y_{i}\right\}}_{i=1}^{N_{c}}$, where $N_{c}$ is the number of input and output data pairs. Algorithm 2, based on continuous learning, aims to learn the ACDN weight parameter $W^{c}$ by solving the following problem:

(8) $$\underset{W^{c}}{\min}Loss\left(W^{c};W^{c-1},D_{c}\right)+\lambda \Omega \left(W^{c}\right)$$

where Loss(W) is a task-specific loss function, $W^{c}$ is the weight parameter at c classes, and $\Omega (W^{c})$ is the regularization based on the L1 or L2 norm to enforce our model $W^{c}$ appropriately. In the case of the ACDN of primary interest, $W^{c}={\left\{W_{l}\right\}}_{l=1}^{L}$ is the weight parameter, which consists of a tenser, where L is the total number of layers of the ACDN.

**Figure 4:**
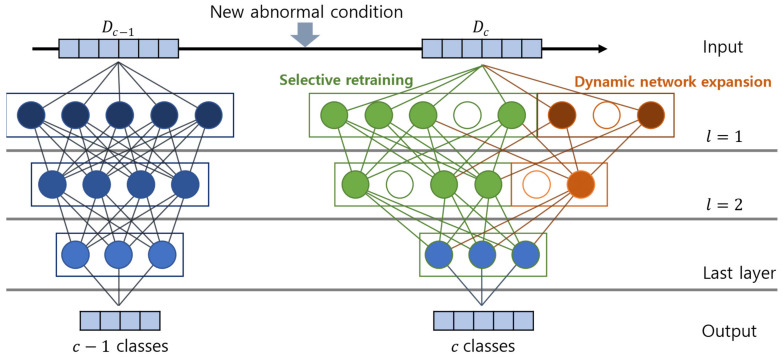
Overview of model optimization methods. When a new abnormal condition is detected in algorithm 1, additional neurons are automatically added to the network to optimize the model.

The ACDN utilizes most of the knowledge obtained from the previous tasks and dynamically extends its capacity when the accumulated knowledge is insufficient to explain the new task. Figure 4 describes the incremental learning process of Algorithm 2, which consists of two parts: selective retraining and dynamic network expansion.

The naivest approach for continuous learning would be to retrain the entire model whenever a new abnormal condition is detected. However, such retraining can be prohibitively expensive for deep neural networks. To address this issue, this study proposes an automatic selective retraining of the model, which retrains only the weights affected by the addition of a new abnormal condition to the dataset. When the first abnormal condition occurs (c=2), the base model of the ACDN is trained with L2 regularization with a typical machine learning model training method. 

Because $W^{c-1}$ remains sparse throughout the incremental learning process in Algorithm 2, the computation cost can be drastically reduced if the subnetwork connected to a new task can be concentrated. Therefore, when an increased number of abnormal conditions c arrives at the ACDN, a sparse linear model is fitted to predict c through the topmost weight parameter of the hidden layer by solving the following problem:

(9) $$\underset{W_{L}^{c}}{\min}Loss\left(W_{L}^{c};W_{1:L-1}^{c-1},D_{c}\right)+\mu {\left\|W_{L}^{c}\right\|}_{1}$$

where $W_{L}^{c}$ is the weight parameter in the Lth layer at c classes and $W_{1:L-1}^{c-1}$ denotes the collection of L−1 weight parameters from the 1st layer to the L−1st layer. μ is the regularization parameter for the sparsity in $W_{L}^{c}$. Before calculating (8), all the weight parameters in $W_{1:L-1}^{c-1}$ are fixed, and $W_{L}^{c}$ is then calculated using L1 regularization to obtain the connection between the last fixed layer and the weight parameters at layer L−1. L1 regularization can promote sparsity in the weight parameters, such that each neuron is connected to only a few neurons in the next layer. After the ACDN on c classes is built, the sparse connection at this layer, $W_{L}^{c}$, which is not zero, is strongly correlated with the new abnormal condition. Specifically, a breadth-first search can be performed on the network, starting from the selected neurons, to identify all the neurons that have paths to the last layer. Subsequently, only the weight parameters of the selected neurons S from all layers, denoted as $W_{1:L,S}$, are trained.

(10) $$\underset{W_{1:L,S}^{c}}{\min}Loss\left(W_{1:L,S}^{c};W_{1:L,S}^{c-1},D_{c}\right)+\mu {\left\|W_{1:L,S}^{c}\right\|}_{2}$$

The element-wise L2 regularizer is employed for training $W_{1:L,S}^{c}$ because sparse connections have already been established at the higher $W_{L}^{c}$ training course. This partial retraining will reduce the computational overhead and help to avoid negative transfer, because the unselected neurons will not be affected by the retraining process. 

Furthermore, additional neurons need to be added to the network to account for the essential features of the new classes. This is known as dynamic network expansion. In conventional incremental learning, a specific number of neurons are added to increased classes regardless of the difficulty of the classification problem, resulting in a suboptimal performance and network capacity usage. To overcome these constraints, a method based on group sparse regularization is proposed to dynamically determine the number of neurons added to each layer when a new class is added, without retraining the network for each class.

Suppose that the lth layer of a network is expanded with K neurons, resulting in the expansion of the two-parameter matrices: $W_{l}^{c}=[W_{l,S}^{c};W_{l}^{K}]$ for the outgoing and incoming layers, where $W_{l}^{K}$ is the expanded weight parameter resulting from the added neurons at the lth layer. Depending on the features of the new abnormal condition, the model does not always require adding in all the K neurons. Therefore, group sparsity regularization on the added parameters is performed as follows:

(11) $$\underset{W_{l}^{K}}{\min}Loss\left(W_{l}^{K};W_{l,S}^{c},D_{c}\right)+\mu {\left\|W_{l}^{K}\right\|}_{1}+\gamma \sum_{g}{\left\|W_{l,g}^{K}\right\|}_{2}$$

where g∈G is a group defined by the incoming weight parameters for each neuron. This group sparsity regularization [16,17] is used to obtain the appropriate number of neurons for a full network, while it is adopted for a partial network in this study. Through group sparsity regularization, the weight parameters deemed unnecessary for training will be eliminated. Consequently, after the dynamic network extension process, the model captures the additional properties not previously represented by $W_{l}^{K}$ to minimize the residual errors, while maximizing the network capacity usage by avoiding the addition of abundant units. 

## 3. Experimental Verification of the Performance of the Proposed Method

In Section 3, the performance of the proposed method is verified using a dataset acquired from a health-monitoring system built into an actual PSH system. The new class identification performance and classification accuracy of the proposed method were thoroughly examined by comparing its results with those of previous methods. The target PSH system is located in South Korea and consists of two 300 MW pump-turbine systems. These two pump turbines have been operating independently since 2007, and a condition-monitoring system with several types of sensors has been deployed in the target PSH system since 2012.

### 3.1. Description of the Target PSH System and Its Condition-Monitoring System

The monitoring system in the target PSH system collects measurement data from the sensors and simultaneously stores them on a database server so that administrators can readily check the conditions of the target PSH system. In the two pump-turbine systems of the PSH, 61 monitoring sensors, which are related to the behavior of real-time PSH, and peripheral devices are installed in the same manner. Because the hydropower turbine is immersed in water and rotates during its operation, the monitoring sensors are mounted on the outer surface of the structure and indirectly monitor the PSH conditions. 

**Figure 5:**
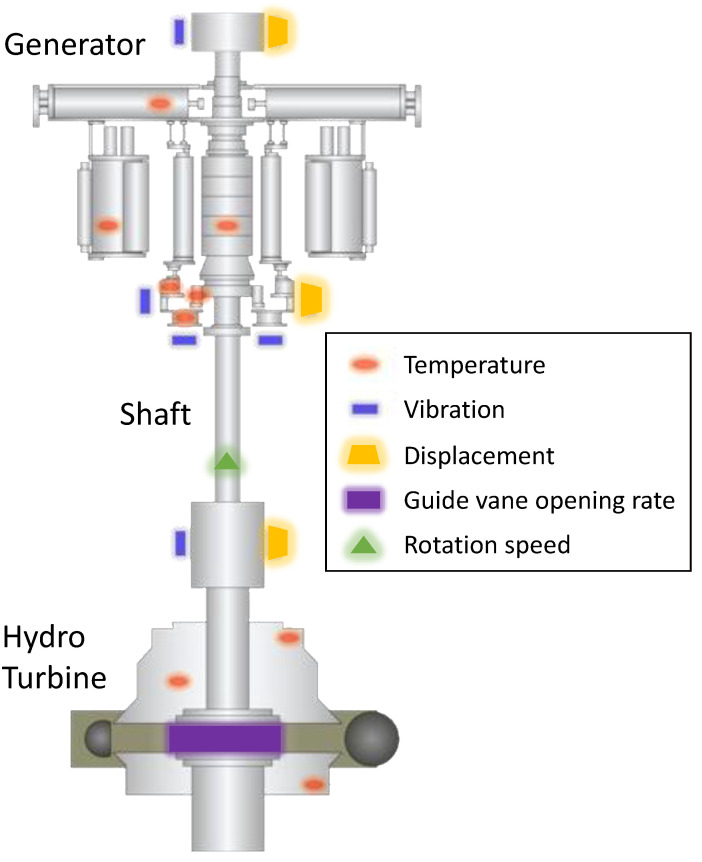
Sensor location of the target PSH-monitoring system.

In this study, the sensors are closely associated with real-time PSH behavior. The data obtained from the 61 sensors were used for a classification of the PSH conditions, including 44 temperature, 9 vibration, 6 displacement, 1 guide vane opening rate, and 1 hydropower turbine rotation speed sensors. Figure 5 presents an overview of the installation locations of the sensors. Table 2 lists the variables and number of monitoring sensors. 

### 3.2. Dataset

In this study, PSH condition-monitoring data from 2016 and 2017 were used. Every year, abnormal conditions occurred up to 5% of the time during the target PSH period. Based on the target PSH-monitoring data, four types of abnormal conditions were identified within this timeframe. As listed in Table 3, two abnormal conditions were observed in the pump state: (1) sequence failure and (2) high vibration. Two abnormal conditions were observed in the turbine state: (1) crashing noise and (2) operating error [18].

The model proposed in this study performed training and testing with the data acquired when the PSH system was operating to select the meaningful data. The data of each sensor used continuously acquired data without preprocessing. To sort the appropriate data from the entire data, the data were collected when the rotation speed of the generator was higher than 299 RPM, which is capable of generating an alternating current with a 60 Hz frequency. Because storing entire monitoring data requires huge space, the monitoring data of the target PSH system are stored with an event-driven method at each monitoring sensor to reduce the storage data size in the database server. Additionally, the sampling rates of the monitoring data of all the sensors were not identical, in order to optimize the data storage. Afterward, the sampling rates of all the sensors were adjusted to 1 Hz through interpolation to synchronize the data acquisition time of all the sensors. For the data interpolation, a step function was used, because an actual monitoring system cannot predict the next monitoring data.

Because the model had not been sufficiently exposed to abnormal condition data, generalizing the algorithm with the monitoring data was challenging for training the machine learning model. Therefore, when unbalanced datapoints were used, the datapoints were adjusted appropriately in advance and compensated for the result depending on the ratio of the actual datapoints after training. To configure the training and validating datapoints in this study, an under-sampling method was used, which reduced the size of the abundant class to balance all the datasets.

Table 4 lists the number of balanced datapoints in each abnormal condition. As abnormal condition #3, which is a sequential failure of the turbine state, had the least number of datapoints, 384 datapoints in other abnormal conditions were randomly sampled using an under-sampling method. Owing to the wide range of summits, the sampling of the normal condition data was performed in a specific range. The normal condition data were randomly sampled 15 days before and after the occurrence of an abnormal condition. The number of normal condition data was set as 1152, three times more than the number of abnormal condition data. 

The constant exposure of PSH to the outdoor environment affects the accuracy of the monitoring sensors. Therefore, outdoor environmental factors were minimized through dataset normalization before the proposed model was trained with the monitoring data. Normalization was performed using the min–max method for 10 days of data collection.

(12) $$o_{m}=\frac{p_{m}-min\left(P\right)}{max\left(P\right)-min\left(P\right)}$$

where $o_{m}$ is the *m*th normalized data, $P=(p_{1},\ldots ,p_{m})$ is the original 10 days data, and $p_{m}$ is the *m*th original data.

### 3.3. Comparison Models for Validation and Model Training 

To validate the proposed method, four conventional machine learning models were used. All the models employed a three-layer network with batch normalization and ReLU activations. The models are summarized as:

- DNN-MTL (reference model): base deep neural networks (DNN) are manually trained for each task separately. MTL indicates “Manually Task Learning.” Without class incrementation, this is the most conventional machine learning model for classification. This model is optimized to have the highest classification accuracy for the data of this study.
- DNN-fine—same architecture as DNN-MTL model, trained for initial tasks and the fine-tuning of the last layer under an increasing number of conditions.
- INN—increment neural network (INN) for each task consistently, based on incremental learning. The most widely used machine learning model for increasing the number of classes.
- ACDN—the proposed model.
- ACDN-1st—base ACDN, applies Algorithm 2 at only the first layer. The computing cost and time are lower than those of the ACDN.

Except for DNN-MTL, all the models were continuously optimized based on pretrained models using the data from the two classes. Furthermore, ACDN and ACDN-1st automatically detected new classes. Because INN and DNN-fine could not recognize a new class automatically, these models were informed of the class increase in the training section. In contrast, the most commonly used classification model, DNN-MTL, was optimized for all the classes so that it could be used as a reference to compare the performances of other models, displaying the best performance on the test dataset.

All the models were coded in the Python programming language with the Pytorch framework [19]. All the models were built and trained using a workstation equipped with an Intel Core i7-9700 processor, Nvidia Force 2060 super 8 GB GPU, and 32 GB of RAM.

For the model optimization, the Adam optimizer [20] was used and the hyperparameters in the algorithm were selected using Bayesian optimization [21]. To prevent overfitting from excessive repetitive training, the error in the training result was checked for each epoch, and the training was stopped if the loss did not decrease in three epochs. The maximum epoch was set to 1000. 

The *F*1 *score* was used as an indicator to evaluate the performance of the proposed model. The precision and recall were calculated for each model and the *F*1 *score* was the harmonic mean of the precision and recall. Fault detection systems are precise in detecting faults and determining whether they are an actual fault, implying that accuracy is measured only for the result wherein the fault detection system determines it to be a fault. Recall is a measure of how well the defect detection system detects actual failure data as a failure without omission. The precision, recall, and *F*1 *score* were calculated as follows: The *F*1 *score* is mostly utilized in applications such as condition monitoring, where the value of false detection is significant.

(13) $$Precision=\frac{TRUE\;detections}{whole\;detections}$$

(14) $$Recall=\frac{TRUE\;detections}{total\;number\;of\;existing\;TRUE}$$

(15) $$F1\;score=2\times \frac{1}{\frac{1}{Precision}+\frac{1}{Recall}}=2\times \frac{Precision\times Recall}{Precision+Recall}$$

### 3.4. Performance Evaluation 

The classification and new class detection performance were evaluated using unknown classes. A sample was randomly selected from an abnormal condition that was not used during the training to create the unknown classes.

To evaluate the new class detection performance for an unknown abnormal class, the proposed ACDN was compared to two conventional methods, (1) SoftMax and (2) Openmax [22], to evaluate its recognition performance for unknown class detection. When the SoftMax probability that an input signal x belonged to a class j was less than a predefined threshold value θ, it was classified as an unknown condition. Here, the value of θ should be adjusted manually for the best classification accuracy, as a higher θ guarantees a better accuracy for new condition detection, while a poorer accuracy for the classification of common conditions. In this study, the SoftMax method was adjusted to θ = 0.7.

Table 5 shows the results of the unknown class detection. In the case of the ACDN, the *F*1 *score* was approximately 99% for all the tests of the total datapoints. This was approximately 23% higher than the SoftMax method with a 0.7 threshold value and 6% higher than the Openmax method. Nevertheless, even with only three data points, the ACDN had an *F*1 *score* of more than 95%. This was more than a 10% improvement over the Openmax result. The results summarized in Table 5 indicate that the new class detection performance of the proposed ACDN was significantly better than that of the conventional method.

**Figure 6:**
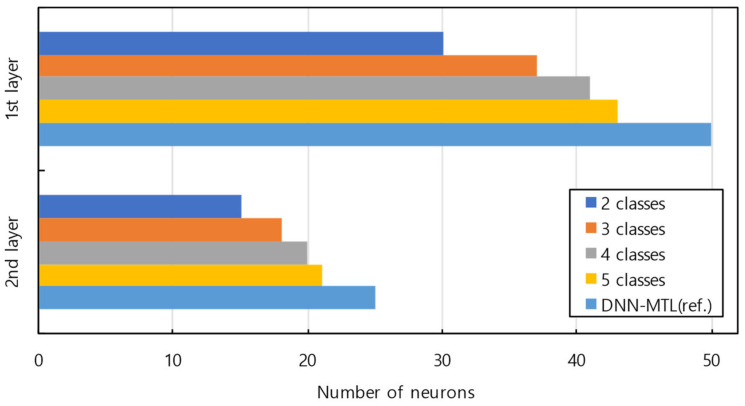
Number of neurons changes in ACDN based on the number of trained classes.

After identifying a new class, the suggested model dynamically adjusted the architecture to cover an increasing number of classes. Figure 6 shows the number of neuron changes in the proposed ACDN with respect to the number of classes. The number of neurons increased slightly with the number of classes and converged. This was the result of efficient feature selection by optimizing the number of neurons in the selective retraining of Algorithm 2 by dropping low-importance neurons. The proposed ACDN consisted of fewer neurons than DNN-MTL, which was trained with a full dataset of five classes and used as a reference model. Owing to the pre-reflection of each condition, the development model could be composed of a minimized number of neurons, as it was continuously learned.

The classification performance of the automatically optimized architecture of the proposed model utilizing algorithm 2 was validated. A few significant metrics for evaluating the average per-class classification of the proposed ACDN are listed in Table 6. Before the test, the ACDN was optimized for the condition monitoring of PSH using two classes: normal and randomly selected abnormal conditions. At this point, the expert checked to see if the learning was proceeding well and obtained an *F*1 *score* of 100% in two classes. Subsequently, whenever the class of the input data increased, the previously trained model was continuously optimized without the guidance of an expert. This process was repeated until the total number of classes reached five. The precision slightly decreased as each class was added. However, all the results consistently demonstrated that the proposed method showed a high performance of > 99% in its precision. Here, all the values of the recall were identically 100%, regardless of the number of classes, because the ACDN did not misdiagnose the normal condition as an abnormal condition. This advantage proved the high reliability of the condition-monitoring performance of the ACDN. The computing time of the ACDN was 0.0076 s per one datapoint of the five-class dataset. Therefore, the developed system is sufficiently applicable for real-time monitoring because it can process sensor data at a field frequency of > 120 Hz of sampling frequency.

**Figure 7:**
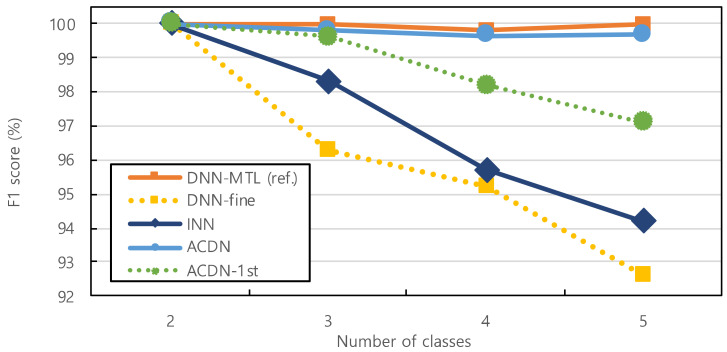
Average per-class accuracy of the models over the number of classes.

Figure 7 shows the average per-class accuracy of the model with respect to the number of classes. To reduce the error, all the models were trained and tested 50 times. Regardless of the number of classes, the ACDN produced results that were as accurate as the DNN-MTL results in the absence of expert intervention. Furthermore, DNN-fine, which simply fine-tuned the last hidden layer of neurons, produced the worst results, confirming that an appropriate number of hidden layers based on the number of classes was essential for enhancing the classification model accuracy. When comparing the ACDN with INN, the selective retraining algorithm and dynamic network extension algorithm outperformed by simply adding hidden layers. Although only the first hidden layer change in ACDN-1st produced a high accuracy, changing the entire model organically was more effective, as in the case of the ACDN.

## 4. Conclusions and Further Discussion

In this study, a PSH condition-monitoring method called the auto-learning and class detection network model (ACDN) was proposed. Unlike previous machine-learning-based structural condition-monitoring methods, the proposed ACDN could detect new abnormal conditions that had not been trained and update and optimize the model itself without human intervention. The proposed ACDN was based on a continuous learning algorithm, which continuously learnt when a new class was detected. By applying actual condition-monitoring data from an actual PSH system, the authors verified that the proposed ACDN detected new abnormal conditions with less than three datapoints for each new abnormal condition, with an *F*1 *score* of 95.89%. Additionally, the *F*1 *score* of the classification could achieve 99.73% with the auto-optimized ACDN, and the computing time for the single input data was only 0.0076 s.

In this study, detection and optimization were accomplished only for a single unknown abnormal condition in the target PSH system. If more than two unknown abnormal conditions occurred, the proposed method detected only a single abnormal condition. Currently, we are focusing on developing a model that can generalize the correlation between the monitoring data from similar structures and abnormal conditions. Additionally, we plan to build an extended machine learning model to support the maintenance of the target PSH system by deriving the problem part from an analysis of the correlation with the abnormal state accumulated from similar structures, such as traditional hydroelectricity, nuclear power plants, and wind power plants, by combining the proposed ACDN with the ongoing research model.

## Figures and Tables

**Table 1:** Typical abnormal conditions occurring in the facilities of a PSH.

Facility	Typical Abnormal Conditions
Runner Draft tube	-Steel wear and leaking -Fatigue stress and cracks
Guide vane	-Efficiency degradation -Operating error-Loosening of bolts and bearing damage
Shaft	-Misalignment -Over-vibration -Distortion and fatigue
Generator	-Low insulation resistance -Shortening and sequence failure -Over-vibration -Overheating and thermal stress

**Table 2:** Physical quantities for monitoring.

Physical Quantities	Sensor Type	Number of Sensors
Temperature (°C)	Resistance temperature detector	44
Vibration (mm/s)	Eddy current proximity sensor	9
Displacement (µm)	Laser displacement sensor	6
Rotation speed (RPM)	Switch sensor	1
Guide vane opening rate (%)	Customized sensor	1

**Table 3:** Selected datapoints of normal and abnormal conditions in 2016 and 2017.

Operation State	Condition	Datapoints	Configuration of an Abnormal Condition
Pump state	Normal	6,856,351	-
	Abnormal #1	13,351	Crashing noise from rupturing residual air in hydropower turbine
	Abnormal #2	488	Operating error due to the malfunction of a guide vane
Turbine state	Normal	6,993,007	-
	Abnormal #3	384	Sequential failure at high output power of a generator
	Abnormal #4	3978	High vibration from cracks at welding points of a hydropower turbine

**Table 4:** Balanced datapoints configuration.

Operation State	Condition	Actual Datapoints	Balanced Datapoints
Pump state	Normal	6,856,351	1152
	Abnormal #1	13,351	384
	Abnormal #2	488	384
Turbine state	Normal	6,993,007	1152
	Abnormal #3	384	384
	Abnormal #4	3978	384

**Table 5:** Comparison of new class detection performance of SoftMax, Openmax, and ACDN methods.

Number of Trained Classes	*F*1 *Score* (%)					
	SoftMax (*θ* = 0.7)		Openmax		ACDN (Proposed)	
	3 DP *	Total	3 DP *	Total	3 DP *	Total
2	76.67	87.42	91.32	97.24	100	100
3	65.21	82.51	87.63	95.52	98.63	99.57
4	52.88	75.16	85.44	93.18	95.89	99.53

* DP = datapoints.

**Table 6:** Average per-class classification evaluation metrics of ACDN.

Number of Classes	2	3	4	5
Precision	100	99.74	99.47	99.48
Recall	100	100	100	100
*F*1 *score*	100	99.87	99.73	99.74

## Data Availability

The data that has been used is confidential.
